# Glutathione Trisulfide Protects Human Epidermal Keratinocytes from UVB-Induced Damage via Restoration of Intracellular Supersulfide Levels

**DOI:** 10.3390/molecules31213871

**Published:** 2026-10-27

**Authors:** Yoshiaki Uchida, Keigo Amari, Masayuki Ito, Toshiya Sato

**Affiliations:** 1 Research and Development Group, Cosmetics Division, Mitsubishi Corporation Life Sciences Limited, 5th Floor, GranTokyo South Tower, 1-9-2 Marunouchi, Chiyoda-ku, Tokyo 100-6605, Japan; 2 Research and Development Division, Mitsubishi Corporation Life Sciences Limited, 1-1-3 Yurakucho, Chiyoda-ku, Tokyo 100-0006, Japantoshiya.sato@mcls-ltd.com (T.S.)

**Keywords:** glutathione trisulfide, GSSSG, supersulfide, NHEK, keratinocyte, NRF2

## Abstract

Ultraviolet B (UVB) irradiation induces oxidative stress and inflammation in keratinocytes, contributing to skin damage and photoaging. Although supersulfides have recently emerged as important regulators of cellular redox homeostasis, their role in UVB-induced skin damage remains poorly understood. In this study, we investigated the effects of glutathione trisulfide (GSSSG), a representative supersulfide, in UVB-exposed normal human epidermal keratinocytes (NHEKs). UVB irradiation significantly reduced cell viability and intracellular supersulfide levels while increasing reactive oxygen species (ROS) production and the expression of IL-1α, prostaglandin E2, and *p21*. Pretreatment with GSSSG significantly restored intracellular supersulfide levels, improved cell viability, suppressed ROS accumulation and inflammatory mediator production, and attenuated *p21* induction. Furthermore, GSSSG significantly promoted nuclear factor erythroid 2-related factor 2 nuclear translocation and increased heme oxygenase-1 production, indicating activation of antioxidant defense pathways. Knockdown of cysteinyl-tRNA synthetase 2 (CARS2), a key enzyme involved in endogenous supersulfide biosynthesis, increased cellular susceptibility to UVB-induced cytotoxicity, whereas GSSSG restored the viability of CARS2-deficient cells. Collectively, these findings suggest that GSSSG protects keratinocytes against UVB-induced oxidative stress, inflammation, and cytotoxicity. This protective activity may be mediated, at least in part, through restoration of intracellular supersulfide levels and activation of the NRF2-mediated antioxidant response.

## 1. Introduction

Ultraviolet B (UVB) radiation is a major cause of epidermal damage because it is readily absorbed by keratinocytes and induces oxidative stress via excessive reactive oxygen species (ROS) production [1,2,3]. Increased ROS production causes DNA photolesions, lipid peroxidation, and protein oxidation, which activate intracellular stress pathways and promote the release of pro-inflammatory cytokines, including IL-1α, IL-6, and TNF-α. These oxidative and inflammatory responses accelerate cellular senescence and impair essential epidermal functions, ultimately contributing to photoaging, disruption of skin barrier integrity, and UVB-induced cell death [4,5,6].

Recent advances in sulfur biology have revealed that reactive sulfur species containing multiple sulfur atoms, collectively referred to as supersulfides, are endogenously produced in humans and other organisms via the activity of biosynthetic enzymes such as cysteinyl-tRNA synthetase 2 (CARS2) [7,8]. Supersulfides participate in a variety of biological processes, including the regulation of oxidative stress, modulation of inflammatory responses, and maintenance of mitochondrial energy metabolism [9,10]. In addition to these systemic functions, recent studies have demonstrated their involvement in cutaneous biology, showing that supersulfides can influence melanogenesis in melanocytes [11]. Owing to their unique chemical reactivity and broad physiological functions, supersulfides are increasingly recognized as important regulators of cellular homeostasis, and interest in their roles in both dermatological and systemic processes has expanded rapidly in recent years. However, the effect of UVB irradiation on intracellular supersulfide levels in human epidermal keratinocytes remains unknown.

Glutathione trisulfide (GSSSG) is a representative member of the supersulfide family and has been characterized by its potent antioxidant and anti-inflammatory properties [12,13]. In addition, GSSSG enhances cellular energy metabolism and exerts cytoprotective effects in various cell types under diverse stress conditions [14]. Notably, GSSSG has been shown to increase intracellular levels of reactive sulfur species [13]. Studies in skin biology have also demonstrated that GSSSG suppresses melanin production in melanocytes [15]. Collectively, these findings establish GSSSG as a biologically active supersulfide with broad potential applications in skin health and disease.

However, the cytoprotective effects of GSSSG against UVB-induced skin damage remain largely unexplored, and the relationship between UVB exposure and intracellular supersulfide levels has not been clarified. Nuclear factor erythroid 2-related factor 2 (NRF2) is a key regulator of cellular antioxidant defense and plays an important role in protecting cells against UVB-induced oxidative damage. Therefore, in addition to evaluating the effects of GSSSG against UVB-induced injury and intracellular supersulfide homeostasis in normal human epidermal keratinocytes (NHEKs), we investigated the involvement of NRF2 signaling in the cytoprotective effects of GSSSG. Furthermore, because CARS2 is a major biosynthetic enzyme for endogenous supersulfides, CARS2 knockdown was employed to determine whether depletion of intracellular supersulfides influences UVB sensitivity and NRF2-mediated antioxidant responses.

## 2. Results

### 2.1. GSSSG Protects NHEK Cells Against UVB

**Figure 1:**
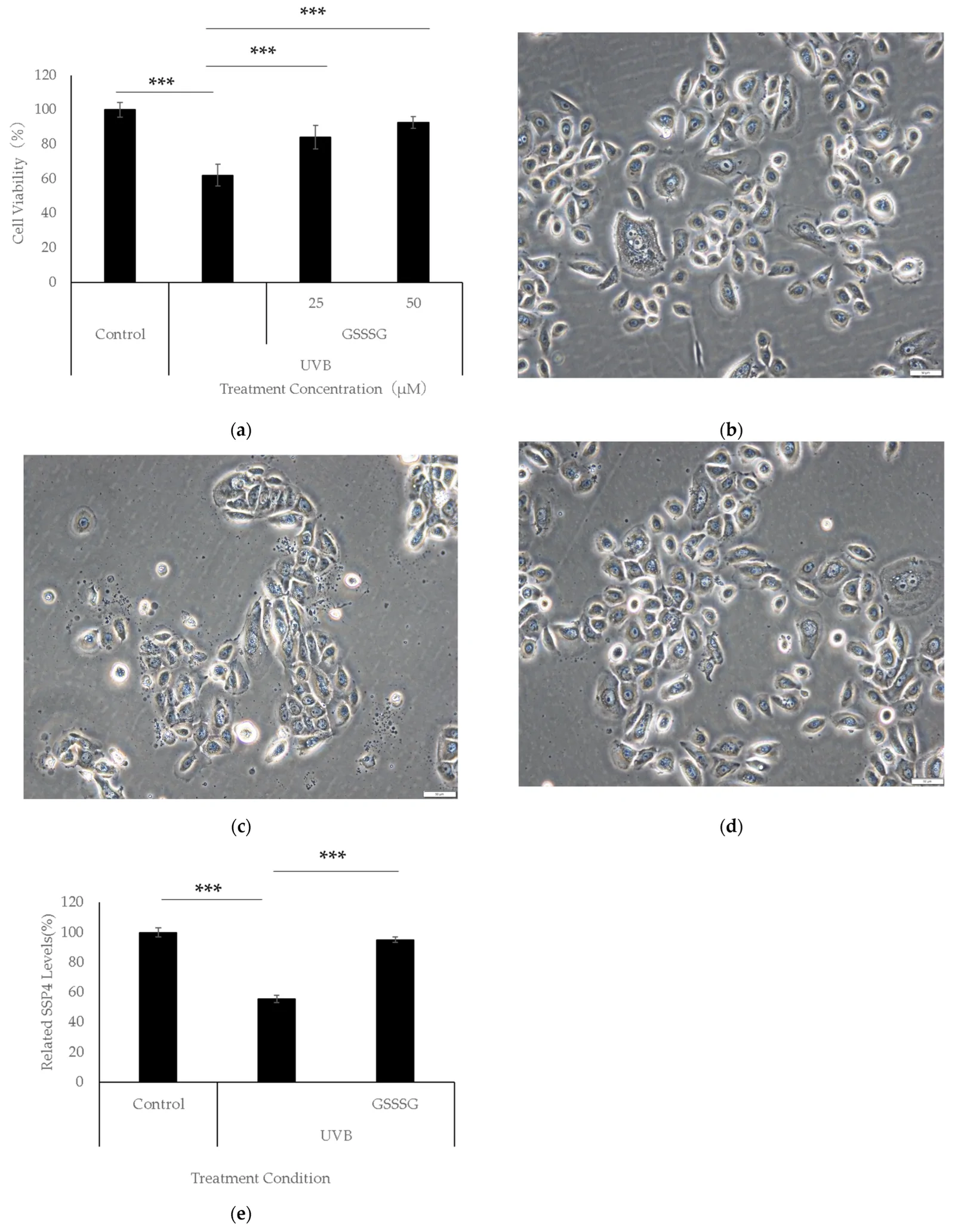
Effects of GSSSG on cell viability and intracellular supersulfide levels in UVB-irradiated NHEKs. NHEK cells were pretreated with GSSSG for 30 min prior to UVB irradiation (20 mJ/cm^2^: the nominal instrument setting). Data are presented as the mean ± standard deviation (SD). Statistical comparisons with the only UVB group were performed using Dunnett’s test (*** *p* < 0.001). (**a**) Cell viability of NHEKs following UVB irradiation with or without GSSSG pretreatment (*n* = 6). (**b**–**d**) Representative microscopic images of control, UVB-irradiated, and GSSSG-pretreated UVB-irradiated NHEKs, respectively. Scale bar was 50 μm. (**e**) Intracellular supersulfide levels in NHEKs following the indicated treatment. GSSSG concentration was 50 μM (*n* = 4).

First, the optimal UVB irradiation dose was determined by evaluating a range of exposure levels, and 20 mJ/cm^2^ was selected for subsequent experiments. Cell viability was assessed following UVB irradiation with or without pretreatment with the indicated concentrations of GSSSG. UVB exposure significantly reduced cell viability compared with that in the control group (Figure 1a). Notably, pretreatment with GSSSG attenuated the UVB-induced decrease in cell viability in a concentration-dependent manner. In contrast, treatment with GSSSG alone in the absence of UVB irradiation did not significantly affect cell viability compared with that in the control group (Supplementary Figure S1a). Morphological examination further revealed that UVB irradiation induced apparent cellular damage in NHEKs. Compared with the control group, UVB-treated cells exhibited increased cell detachment and accumulation of cellular debris, indicative of cytotoxicity. These UVB-induced morphological alterations were markedly suppressed by GSSSG pretreatment. Cells treated with GSSSG retained a more intact cellular morphology and exhibited fewer detached cells and less cellular debris than cells exposed to UVB alone (Figure 1b–d). Furthermore, UVB irradiation significantly reduced intracellular supersulfide levels compared with those in the control group. This reduction was significantly reversed by GSSSG pretreatment, indicating that GSSSG restored intracellular supersulfide levels in UVB-exposed NHEKs (Figure 1e). Interestingly, GSSSG treatment alone significantly increased intracellular supersulfide levels compared with those in the control group, indicating that exogenous GSSSG can enhance intracellular supersulfide homeostasis under non-stressed conditions (Supplementary Figure S1b).

### 2.2. GSSSG Protects Against UVB-Induced Oxidative Stress and Inflammation

**Figure 2:**
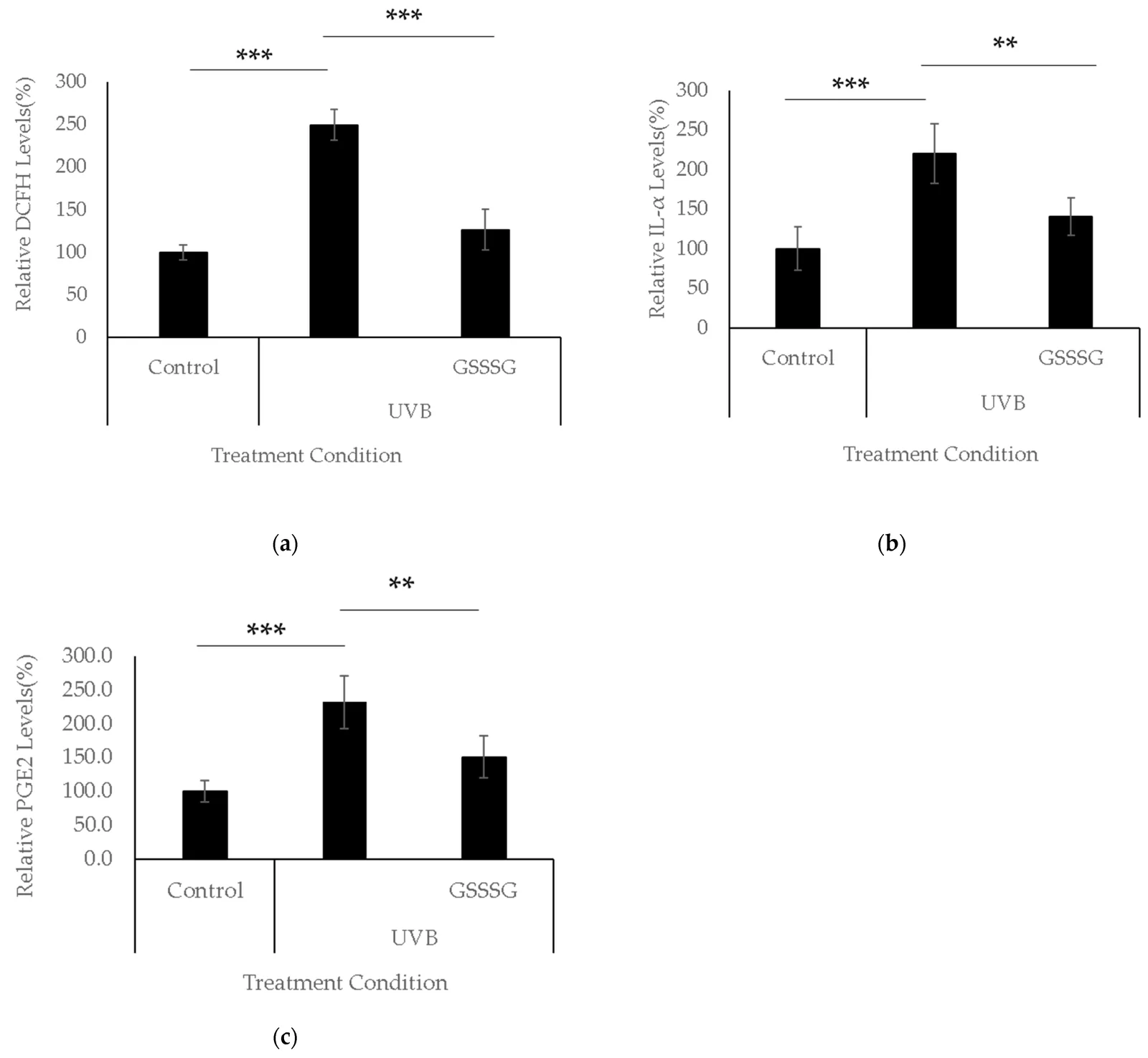
Effects of 50 μM GSSSG on UVB-induced oxidative stress and inflammatory responses in NHEKs. NHEKs were pretreated with 50 μM GSSSG for 30 min prior to UVB irradiation (20 mJ/cm^2^: the nominal instrument setting). Data are presented as the mean ± standard deviation (SD). Statistical comparisons with the only UVB group were performed using Dunnett’s test (** *p* < 0.01, *** *p* < 0.001). (**a**) Intracellular ROS levels following the indicated treatments (*n* = 5). (**b**,**c**) Expression levels of inflammatory responses (*n* = 4).

Next, we examined the effects of GSSSG on UVB-induced oxidative stress and inflammatory responses in NHEKs. Intracellular ROS levels were significantly increased by UVB irradiation compared with those in the control group. Pretreatment with GSSSG significantly suppressed this UVB-induced increase in ROS levels (Figure 2a). Similarly, UVB irradiation significantly increased the levels of the inflammatory mediators IL-1α and PGE_2_ relative to the control group. However, GSSSG pretreatment significantly attenuated the UVB-induced increases in both IL-1α and PGE_2_ levels (Figure 2b,c).

### 2.3. Effects of GSSSG on NRF2-Mediated Antioxidant Signaling

**Figure 3:**
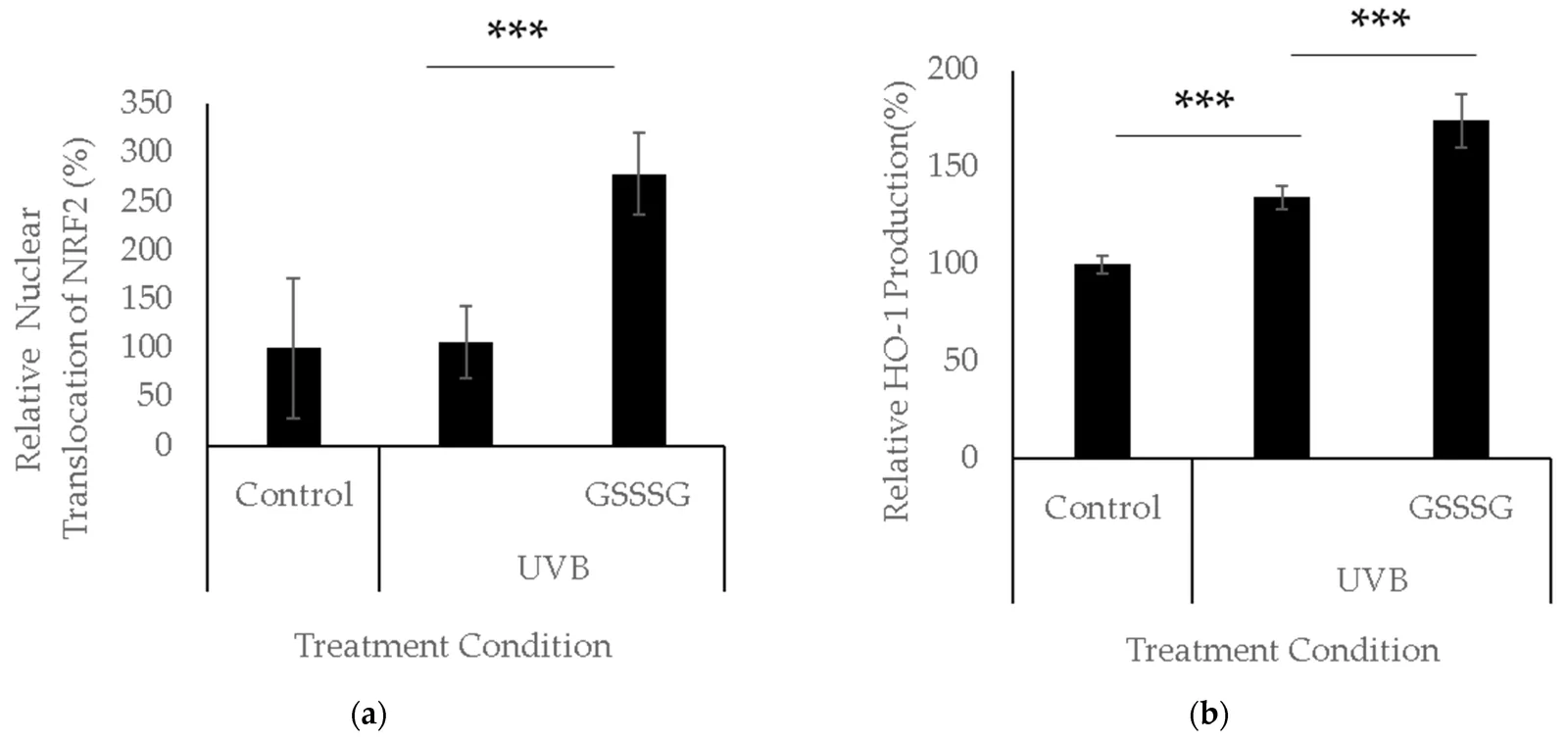
Effects of 50 μM GSSSG on the NRF2 pathway in UVB-exposed NHEKs. NHEKs were pretreated with 50 μM GSSSG for 30 min prior to UVB irradiation (20 mJ/cm^2^: the nominal instrument setting). Data are presented as the mean ± standard deviation (SD). Statistical comparisons with the only UVB group were performed using Dunnett’s test (*** *p* < 0.001). (**a**) Nuclear translocation of NRF2 following the indicated treatments (*n* = 6). (**b**) HO-1 expression levels following the indicated treatments (*n* = 4).

Next, we investigated whether the protective effects of GSSSG were associated with activation of the NRF2-mediated antioxidant defense pathway. As shown in Figure 3a, nuclear translocation of NRF2 was significantly increased in the GSSSG + UVB group compared with the UVB group. Consistent with this finding, the expression of HO-1, a downstream target of NRF2, was also significantly increased by GSSSG treatment (Figure 3b and Figure S2a). These results suggest that GSSSG treatment is associated with activation of NRF2 signaling in UVB-exposed NHEKs. This response may contribute to the observed cytoprotective effects of GSSSG, including preservation of cell viability and suppression of intracellular ROS accumulation.

### 2.4. GSSSG Attenuates UVB-Induced Cellular Senescence Marker in NHEKs

**Figure 4:**
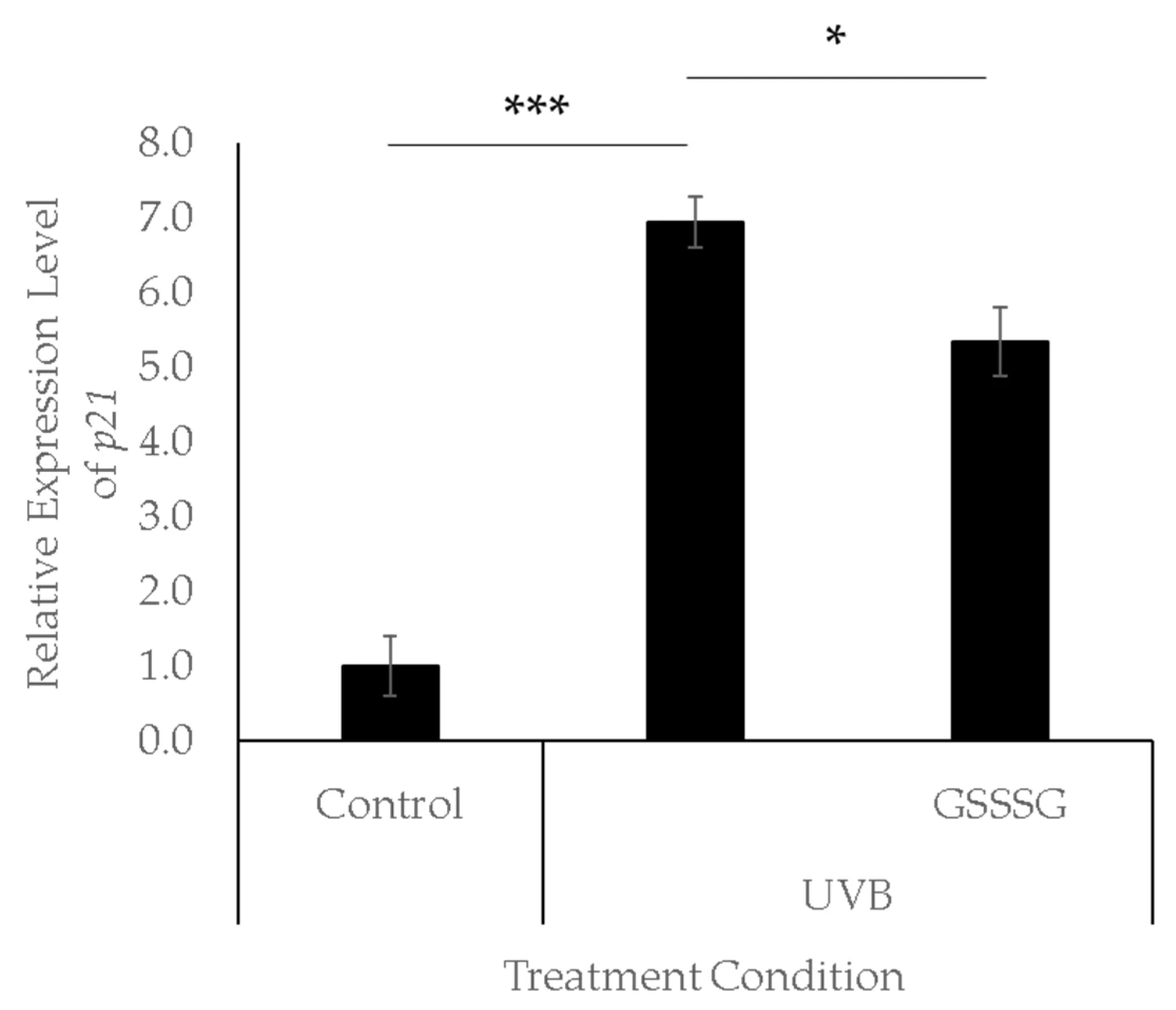
Effects of 50 μM GSSSG on UVB-induced *p21* expression in NHEKs. NHEKs were pretreated with 50 μM GSSSG for 30 min prior to UVB irradiation (20 mJ/cm^2^: the nominal instrument setting). Data are presented as the mean ± standard deviation (SD). Statistical comparisons with the only UVB group were performed using Dunnett’s test (* *p* < 0.05, *** *p* < 0.001). Relative *p21* mRNA expression levels following the indicated treatments (*n* = 4).

We next examined the effects of GSSSG on the expression of senescence-associated markers. As shown in Figure 4, UVB irradiation significantly increased the mRNA expression of p21 compared with that in the control group. Notably, GSSSG treatment significantly attenuated this UVB-induced increase in *p21* expression. As p21 is a key regulator of cell cycle arrest and cellular senescence, these findings suggest that GSSSG may mitigate senescence-associated responses induced by UVB irradiation in NHEKs (Figure 4).

### 2.5. Effects of CARS2 Knockdown on UVB Sensitivity and the Protective Activity of GSSSG

**Figure 5:**
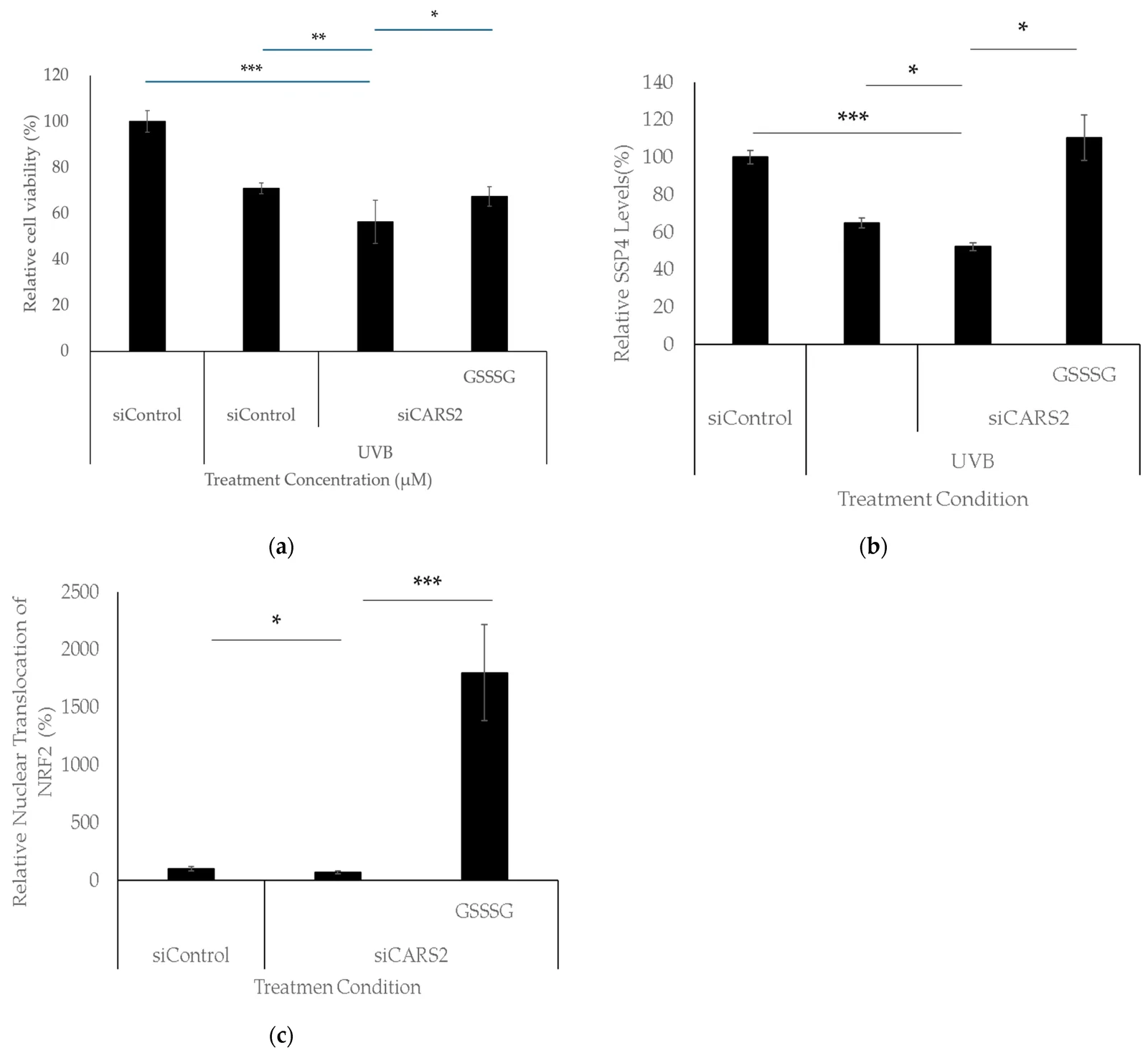
Effects of CARS2 knockdown on UVB-induced cytotoxicity and the protective activity of 50 μM GSSSG in NHEKs. NHEKs were transfected with siControl or siCARS2 for 16 h and incubated for 24 h after changing the medium. Following incubation with the indicated reagents, cells were exposed to UVB irradiation (20 mJ/cm^2^; the nominal instrument setting). Cell viability was assessed 24 h after UVB exposure. Data are presented as the mean ± SD. Statistical comparisons with the siCARS2 + UVB group were performed using Dunnett’s test (* *p* < 0.05, ** *p* < 0.01, *** *p* < 0.001). (**a**) Relative cell viability of control siRNA and CARS2 siRNA cells following UVB irradiation in the presence or absence of GSSSG (*n* = 6). (**b**) Relative intracellular supersulfide levels of control siRNA and CARS2 siRNA cells following UVB irradiation and GSSSG treatment (*n* = 5). (**c**) Nuclear translocation of NRF2 (*n* = 4). Statistical comparisons with the siCARS2 group were performed using Dunnett’s test (* *p* < 0.05, *** *p* < 0.001).

Because UVB irradiation reduced intracellular supersulfide levels (Figure 1e), we next investigated whether endogenous supersulfide production contributes to cellular resistance against UVB-induced damage. To this end, CARS2, a key enzyme involved in endogenous supersulfide biosynthesis, was silenced using siRNA. Downregulation of CARS2 expression by siCARS2 was confirmed by RT-qPCR (Figure S3). As shown in Figure 5a, CARS2 knockdown significantly increased the susceptibility of NHEKs to UVB irradiation, resulting in a marked decrease in cell viability compared with that in the control siRNA group. Notably, treatment with GSSSG significantly restored cell viability in CARS2-deficient cells to levels comparable to those observed in the control group. Moreover, GSSSG treatment significantly restored intracellular supersulfide levels in CARS2-deficient cells (Figure 5b). *CARS2* knockdown alone did not significantly affect cell viability in the absence of UVB irradiationd. These findings suggest that endogenous supersulfides contribute to the protection of NHEKs against UVB-induced cytotoxicity. Furthermore, the ability of GSSSG to restore cell viability in CARS2-deficient cells indicates that exogenous supplementation can, at least partially, compensate for impaired endogenous supersulfide production. To investigate the potential relationship between supersulfide deficiency and increased sensitivity to UVB irradiation, NRF2 nuclear translocation was evaluated. As shown in Figure 5c, NRF2 nuclear translocation was significantly reduced in the siCARS2 group compared with the siControl group (Figure S2b). In contrast, GSSSG treatment significantly increased NRF2 nuclear translocation in CARS2-knockdown cells.

## 3. Discussion

In the present study, we demonstrated that GSSSG protects NHEKs against UVB-induced cellular damage. Pretreatment with GSSSG restored cell viability, reduced intracellular ROS accumulation, suppressed the production of inflammatory mediators, and attenuated the expression of the senescence-associated marker *p21*. Furthermore, GSSSG restored intracellular supersulfide levels that had been reduced by UVB irradiation and activated the NRF2-mediated antioxidant defense pathway. Collectively, these findings suggest that GSSSG exerts cytoprotective effects against UVB-induced damage via restoration of intracellular supersulfide homeostasis and enhancement of antioxidant defense mechanisms.

Oxidative stress is one of the primary mechanisms underlying UVB-induced skin damage [16,17,18]. In the present study, UVB irradiation significantly increased intracellular ROS levels, whereas GSSSG treatment effectively suppressed ROS accumulation (Figure 2). These findings are consistent with the observed improvement in cell viability and suggest that the antioxidant properties of GSSSG contribute to its protective effects against UVB-induced cytotoxicity. Several mechanisms may account for the antioxidant effects of GSSSG. First, GSSSG significantly promoted NRF2 nuclear translocation and increased HO-1 expression. NRF2 is a master regulator of cellular antioxidant defense and induces the expression of numerous cytoprotective genes in response to oxidative stress [19,20]. Therefore, activation of the NRF2/HO-1 pathway may contribute to the reduction in intracellular ROS levels observed following GSSSG treatment. Supersulfides reportedly promote NRF2 signaling and play important roles in the maintenance of cellular redox homeostasis [21,22]. Consistent with these findings, the present study demonstrated that GSSSG activates the NRF2 signaling pathway in UVB-exposed keratinocytes. Second, GSSSG itself possesses potent antioxidant activity. GSSSG can directly modulate cellular redox status and protect cells against oxidative stress-induced damage [15]. In addition, GSSSG substantially restored intracellular supersulfide levels that had been reduced by UVB irradiation. Supersulfides exhibit strong nucleophilic and radical-scavenging properties and are increasingly recognized as important endogenous antioxidants [23]. Therefore, restoration of intracellular supersulfide levels by GSSSG may further enhance the antioxidant capacity of keratinocytes. Collectively, these findings suggest that GSSSG exerts antioxidant effects via multiple complementary mechanisms, including activation of the NRF2/HO-1 pathway, direct antioxidant activity, and restoration of intracellular supersulfide homeostasis. Notably, in the absence of UVB irradiation, GSSSG treatment significantly increased intracellular supersulfide levels, whereas no significant effect on cell viability was observed (Figure S1). These findings indicate that GSSSG itself does not directly promote cell survival under basal conditions but acts as a source of intracellular supersulfides. Therefore, the protective effects observed under UVB exposure are unlikely to be explained by a simple increase in basal cell viability and may instead be associated with the restoration of intracellular supersulfide homeostasis.

In addition to oxidative stress, inflammation is a major contributor to UVB-induced skin damage. Keratinocytes exposed to UVB produce various inflammatory mediators, including IL-1α and PGE_2_, which contribute to epidermal inflammation and photoaging [24,25]. In the present study, UVB irradiation significantly increased the production of IL-1α and PGE_2_, whereas GSSSG treatment effectively suppressed their induction. The anti-inflammatory effects of GSSSG may be closely linked to its ability to attenuate oxidative stress. Excessive ROS generation is a well-established upstream trigger of inflammatory signaling pathways, and the reduction in ROS levels observed following GSSSG treatment likely contributed to the suppression of inflammatory mediator production. In addition, supersulfides have been reported to regulate inflammatory responses in a variety of biological systems [26,27,28]. Therefore, restoration of intracellular supersulfide levels may represent an additional mechanism by which GSSSG suppresses UVB-induced inflammatory responses.

Another important finding of the present study is the involvement of intracellular supersulfides in the cellular response to UVB irradiation. We demonstrated that UVB exposure significantly reduced intracellular supersulfide levels in NHEKs. Previous studies have reported that intracellular supersulfide levels decrease under various stress conditions, including aging, suggesting that supersulfide depletion may be associated with impaired cellular homeostasis [29,30]. Moreover, hair contains endogenous supersulfides that function as protective molecules against UV-induced oxidative stress [31]. Given the potent antioxidant and anti-inflammatory properties of supersulfides, the reduction observed following UVB exposure may reflect their consumption as part of the cellular defense response to UVB-induced oxidative stress. To further investigate the physiological importance of intracellular supersulfides, we established a supersulfide-deficient cellular model by knocking down CARS2, a key enzyme involved in endogenous supersulfide biosynthesis [32,33]. CARS2 knockdown significantly increased the susceptibility of keratinocytes to UVB-induced cytotoxicity, resulting in a greater reduction in cell viability than that observed in control cells. These findings indicate that endogenous supersulfides contribute to cellular resistance against UVB-induced damage and play an important role in maintaining keratinocyte viability under conditions of oxidative stress. Interestingly, GSSSG treatment restored cell viability and intracellular supersulfide levels even in CARS2-deficient cells (Figure 5). As GSSSG significantly increased intracellular supersulfide levels, this protective effect may be attributable, at least in part, to restoration of intracellular supersulfide homeostasis, and exogenous GSSSG was able to restore the measured endpoints despite CARS2-targeting siRNA treatment.

From an application perspective, the ability of GSSSG to attenuate UVB-induced oxidative stress, inflammation, and cytotoxicity suggests its potential utility as a preventive skincare ingredient for protecting the skin against environmental stressors. Because chronic UV exposure is a major contributor to photoaging and skin damage, maintenance of intracellular supersulfide homeostasis through GSSSG supplementation may support epidermal defense mechanisms and help preserve skin homeostasis. Although the present findings were obtained using an in vitro model and further studies, including clinical investigations, are required, GSSSG may represent a promising candidate for cosmetic applications related to UV care and the prevention of photoaging-associated skin alterations.

One limitation of the present study is that UVB irradiation was performed in HuMedia-KG2 culture medium rather than in a colorless buffer such as HBSS. Although a previous keratinocyte UVB study has employed similar irradiation protocols without explicitly describing medium replacement prior to irradiation, UVB transmission may be partially attenuated by the culture medium [34]. Therefore, the actual UVB dose reaching the cells may differ from the nominal irradiation dose. Although irradiance was measured using a radiometer under the same experimental conditions, a certain degree of influence of the culture medium on UVB exposure cannot be excluded. This should be considered when interpreting the results and comparing UVB doses across different studies. Another limitation of this study is that all experiments were conducted using NHEKs derived from a single donor. Thus, the potential influence of donor-to-donor variability was not evaluated. Another limitation of this study is that the effects of GSSSG were not directly compared with those of other glutathione species, such as GSH or GSSG. Because GSH and GSSG also possess antioxidant properties, they may be expected to confer a certain degree of protection against UVB-induced cellular damage. However, previous studies have reported that GSH and GSSG do not significantly affect intracellular supersulfide levels [14,15]. Therefore, although the present findings demonstrate the biological activity of GSSSG, further studies are required to determine the extent to which the observed effects are attributable to its unique supersulfide-related properties.

Although the present findings provide initial evidence for the protective effects of GSSSG against UVB-induced cellular damage and suggest its potential for skin protection, the results were obtained using a submerged NHEK monoculture system. Therefore, the translational implications should be considered preliminary. Further studies using more complex skin models, as well as clinical investigations, are required to confirm and extend these observations.

Taken together, these findings suggest that maintenance of intracellular supersulfide levels is an important component of the epidermal defense system against UVB-induced damage and that the ability of GSSSG to replenish intracellular supersulfides represents one of the key mechanisms underlying its cytoprotective activity.

## 4. Materials and Methods

### 4.1. Cell Culture and Treatment

NHEKs were obtained from Kurabo (a single donor, KK-4009, Osaka, Japan) and cultured in HuMedia-KG2 medium (TKK-2150S, Kurabo) supplemented with the Cell Proliferation Additive Set (KK-6150, Kurabo), which contains growth supplements and antibiotics (50 μg/mL gentamicin and 50 ng/mL amphotericin B) recommended by the manufacturer for NHEK culture. Cells were maintained at 37 °C in a humidified incubator containing 5% CO_2_.

### 4.2. Determination of UVB and GSSSG Concentrations

A preliminary experiment was conducted to determine an appropriate UVB dose. UVB irradiation was performed using a UVP Crosslinker CL-3000M (Analytik Jena, Jena, Germany; peak wavelength, 302 nm). This device incorporates an internal UV sensor and automatically terminates irradiation when the preset UV dose is reached. Cells were exposed to the indicated UVB doses (10, 20, or 40 mJ/cm^2^; the nominal instrument setting). UVB irradiation was performed in the presence of HuMedia-KG2 culture medium (pink color), and the medium was not replaced before or after irradiation. The medium volume during irradiation was 100 μL/well in a 96-well plate. To avoid attenuation by the plate cover, UVB irradiation was performed without the plate lid. Exposure to 40 mJ/cm^2^ resulted in a marked reduction in cell viability, whereas 20 mJ/cm^2^ reduced cell viability to approximately 60%. Therefore, 20 mJ/cm^2^ was selected for all subsequent experiments. To characterize the actual UVB exposure received by the cells, irradiance at the cell layer was measured using a UV radiometer (UV-19MW, ASONE, Osaka, Japan) under the same experimental conditions. The measured irradiance was approximately 1000 μW/cm^2^. Based on the irradiation time required to achieve the preset dose of 20 mJ/cm^2^ (7 s), the UVB dose reaching the cell layer was estimated to be approximately 7 mJ/cm^2^ (the estimated dose reaching the cell layer). In the present study, untreated cells served as the control group.

The concentrations of GSSSG (20 and 50 μM) were selected based on preliminary MTT assays showing no significant cytotoxicity at concentrations up to 200 μM. Because the experiments involved co-treatment with UVB irradiation, concentrations well below the cytotoxicity threshold were used.

### 4.3. Cell Viability Assay

Cell viability was assessed using the 3-(4,5-dimethylthiazol-2-yl)-2,5-diphenyltetrazolium bromide (MTT; Wako, Osaka, Japan) assay, as previously described [35], with minor modifications. NHEKs were seeded at a density of 1 × 10^4^ cells per well in 96-well plates and incubated at 37 °C in a humidified atmosphere containing 5% CO_2_. After 16 h, the cells were pretreated with GSSSG for 30 min and subsequently exposed to UVB irradiation (20 mJ/cm^2^). The cells were then cultured for an additional 24 h under the same conditions. Following incubation, the culture medium was removed, and 100 μL of fresh medium containing 0.5 mg/mL MTT was added to each well. After incubation for 4 h at 37 °C in the dark, 10% sodium dodecyl sulfate (SDS; Wako) was added to each well to dissolve the formazan crystals, and the plates were further incubated for 16 h at 37 °C. Absorbance was measured at 570 nm using a Varioskan LUX microplate reader (Thermo Fisher Scientific, Waltham, MA, USA).

### 4.4. Detection of Intracellular Supersulfide Levels

Intracellular supersulfide levels were measured using Sulfane Sulfur Probe 4 (SSP4; Dojindo, Kumamoto, Japan) according to the manufacturer’s instructions. SSP4 is a fluorescent probe that detects intracellular sulfane sulfur species, including persulfides and polysulfides. NHEKs were seeded in black 96-well plates (Thermo Fisher Scientific) at a density of 1 × 10^4^ cells per well and cultured at 37 °C in a humidified atmosphere containing 5% CO_2_. After 16 h of incubation, the cells were treated with GSSSG and UVB as described above. Following treatment, the cells were washed with serum-free Dulbecco’s modified Eagle’s medium (Wako) and incubated with 20 μM SSP4 working solution for 15 min at 37 °C in a humidified atmosphere containing 5% CO_2_. Thereafter, the cells were washed twice with phosphate-buffered saline (PBS; Wako), and fluorescence intensity was measured using a microplate reader (Thermo Fisher Scientific) with excitation and emission wavelengths of 482 and 515 nm, respectively. SSP4 fluorescence intensity was normalized to Hoechst fluorescence intensity (Dojindo) to account for differences in cell number among wells.

### 4.5. ROS Assay

Intracellular ROS levels were measured according to the manufacturer’s instructions (Dojindo) and a previous article [36]. NHEKs were seeded in black 96-well plates at a density of 1 × 10^4^ cells per well and cultured under standard conditions. The cells were pretreated with GSSSG and subsequently exposed to UVB irradiation as described above. Following treatment, the culture medium was removed, and the cells were washed twice with Hank’s balanced salt solution (HBSS; Thermo Fisher Scientific). The 10 μmol/L DCFH-DA working solution was then added, and the cells were incubated for 30 min at 37 °C in a humidified atmosphere containing 5% CO_2_. After incubation, the working solution was removed, and the cells were washed once with HBSS. Fresh HBSS was added to each well, and fluorescence intensity was measured using a Varioskan LUX microplate reader (Thermo Fisher Scientific) with excitation and emission wavelengths of 490 and 510 nm, respectively.

### 4.6. Measurement of Protein Levels

The concentrations of interleukin-1α (IL-1α) and prostaglandin E_2_ (PGE_2_) in culture supernatants were quantified using commercially available enzyme-linked immunosorbent assay kits according to the manufacturers’ instructions (IL-1α, ab178008; PGE_2_, ab316263; Abcam, Cambridge, UK). Intracellular HO-1 levels were also measured in cell lysates using an ELISA kit according to the manufacturer’s instructions (ab207621; Abcam, Cambridge, UK). NHEKs were seeded at a density of 1 × 10^5^ cells per well and allowed to attach overnight. The cells were pretreated with GSSSG, subsequently exposed to UVB irradiation (20 mJ/cm^2^), and then incubated for 24 h at 37 °C in a humidified atmosphere containing 5% CO_2_. Total cellular protein was quantified using a bicinchoninic acid (BCA) protein assay kit (Thermo Fisher Scientific). The concentrations of IL-1α, PGE_2_, and HO-1 were normalized to the total protein content of the corresponding samples.

### 4.7. RNA Extraction, cDNA Synthesis, and Real-Time PCR

Total RNA was extracted from cells using the RNeasy Mini Kit (Qiagen, Hilden, Germany) according to the manufacturer’s instructions. Purified RNA was eluted in RNase-free water (Wako, Osaka, Japan), and its concentration and purity were assessed using a NanoDrop 2000 spectrophotometer (Thermo Fisher Scientific). First-strand complementary DNA (cDNA) was synthesized from total RNA using the High-Capacity cDNA Reverse Transcription Kit (Thermo Fisher Scientific) according to the manufacturer’s protocol. Quantitative real-time PCR (RT-qPCR) was performed using SYBR Green Supermix (Bio-Rad, Hercules, CA, USA). Relative mRNA expression levels were calculated using the 2^−ΔΔCt^ method with β-actin as the reference gene. Amplification was performed at 95 °C for 30 s, followed by 40 cycles of 95 °C for 10 s and 60 °C for 20 s. Melting curve analysis was subsequently performed from 65 °C to 95 °C with 0.5 °C increments every 5 s. Primer sequences used for RT-qPCR are shown below.

*p21*
  Forward primer: GAACTTCGACTTTGTCACCGAGAC
  Reverse primer: TGGAGTGGTAGAAATCTGTCATGCT
  β-actin
  Forward primer: TGG CAC CCA GCA CAA TGA A
  Reverse primer: CTA AGT CAT AGT CCG CCT AGA AGC A

### 4.8. siRNA Transfection

Human CARS2-targeting siRNA was obtained from Thermo Fisher Scientific (Cat. No. s35866). MISSION^®^ siRNA Universal Negative Control (Sigma-Aldrich, St. Louis, MO, USA) was used as a non-targeting control. NHEKs were transfected with 50 nM siRNA using Lipofectamine RNAiMAX Transfection Reagent (Invitrogen, Carlsbad, CA, USA) according to the manufacturer’s instructions. After 16 h of transfection, the culture medium was replaced with fresh medium, and the cells were incubated for an additional 24 h. The medium was then replaced with fresh medium containing the indicated reagents, and the cells were further incubated for 24 h. Subsequently, the cells were treated with GSSSG and exposed to UVB irradiation. Cell viability and intracellular supersulfide levels were evaluated as described above.

### 4.9. Immunofluorescence Staining

Cells were seeded in a 96-well black plate at a density of 1 × 10^4^ cells/well and cultured overnight. The following day, cells were treated with the indicated samples and incubated for an additional 4 h. After treatment, cells were fixed with 4% paraformaldehyde (Wako) for 20 min at room temperature. The fixed cells were washed with PBS and blocked for 90 min at room temperature in blocking buffer. Cells were then incubated overnight at 4 °C with an anti-NRF2 rabbit antibody (Cell Signaling Technology, Danvers, MA, USA) diluted 1:500. After washing with wash buffer, cells were incubated for 2 h at room temperature with Alexa Fluor 488-conjugated goat anti-rabbit secondary antibody (Thermo Fisher Scientific) diluted 1:500 and Hoechst solution (Dojindo) diluted 1:1000. The cells were then washed with wash buffer and maintained in PBS for fluorescence imaging. Fluorescence images were acquired from a single field located at the center of each well using an ECLIPSE Ts2R-FL inverted fluorescence microscope (Nikon Corporation, Tokyo, Japan) and NIS-Elements software (Ver.5, Nikon Corporation). NRF2 nuclear translocation was quantified by calculating the ratio of nuclear NRF2 fluorescence intensity to cytoplasmic NRF2 fluorescence intensity. Nuclear regions were defined based on Hoechst staining. The obtained values were normalized to the corresponding control group and expressed as percentages.

### 4.10. Statistical Analysis

Statistical analyses were conducted using EZR software (version 4.1.2; Saitama Medical Center, Saitama, Japan). Data are presented as the mean ± standard deviation (SD) from a minimum of three independent experiments. Differences among groups were evaluated using one-way analysis of variance (ANOVA) followed by Dunnett’s multiple-comparison test, with the UVB-alone group designated as the reference group. Statistical significance was defined as follows: * *p* < 0.05, ** *p* < 0.01, and *** *p* < 0.001. Comparisons between two groups were performed using Student’s *t*-test. Statistical significance was defined as follows: * *p* < 0.05, ** *p* < 0.01, and *** *p* < 0.001.

### 4.11. GSSSG Preparation

GSSSG was supplied by Mitsubishi Corporation Life Sciences Limited (Tokyo, Japan). Purity analysis by high-performance liquid chromatography indicated a purity of 96–97%. GSSSG powder was stored at −80 °C until use. For each experiment, GSSSG was freshly prepared immediately before use and diluted to the desired concentration in culture medium.

## 5. Conclusions

In conclusion, this study demonstrates that GSSSG attenuates UVB-induced damage in human epidermal keratinocytes via antioxidant and anti-inflammatory mechanisms, accompanied by the restoration of intracellular supersulfide levels. Collectively, the findings of this study highlight the key role of intracellular supersulfide homeostasis in the epidermal defense response to UVB-induced damage and suggest that GSSSG has potential as a bioactive ingredient for the prevention of photoaging and the maintenance of skin health.

## Data Availability

The data presented in this study are available upon request from the corresponding author.

## Supplementary Materials

The following supporting information can be downloaded at: https://www.mdpi.com/article/10.3390/molecules31213871/s1, Figure S1: Effects of GSSSG alone on cell viability and intracellular supersulfide levels in NHEKs. NHEKs were treated with 50 μM GSSSG in the absence of UVB irradiation. Data are presented as the mean ± standard deviation (SD). Statistical comparisons with the control group were performed using Student’s *t*-test (*** *p* < 0.001). (a) Cell viability determined by the MTT assay. No significant difference was observed between the control and GSSSG-treated groups. (b) Intracellular supersulfide levels. GSSSG treatment significantly increased intracellular supersulfide levels compared with those in the control group; Figure S2: Representative images of NRF2 nuclear translocation in NHEKs. (a) Representative immunofluorescence images showing NRF2 localization in control, UVB-irradiated, and UVB + GSSSG-treated NHEKs. NRF2 is shown in green, and nuclei were stained with Hoechst (blue). Merged images are shown to visualize NRF2 nuclear translocation. Scale bar = 25 μm. (b) Representative immunofluorescence images showing NRF2 localization in siControl-, siCARS2-, and siCARS2 + GSSSG-treated NHEKs following UVB irradiation. Figure S3. Validation of CARS2 knockdown in NHEKs. CARS2 mRNA expression was measured by RT-qPCR after transfection with control siRNA (siControl) or CARS2-targeting siRNA (siCARS2). Relative CARS2 mRNA levels were normalized to β-actin and expressed relative to the siControl group. Data are presented as mean ± SD (*n* = 3). Statistical significance was determined using Student’s *t*-test. *** *p* < 0.001 vs. siControl.
