# Supplementary material for: Glutathione Trisulfide Protects Human Epidermal Keratinocytes from UVB-Induced Damage via Restoration of Intracellular Supersulfide Levels
Source: Molecules. 2026 Oct 27;31(21):3871. doi: 10.3390/molecules31213871 (PMC13765222; doi:10.3390/molecules31213871)
Supplement: Supplementary file 1 [file molecules-31-03871-s001.zip › molecules-4595685-supplementary.pdf]

Figure S1. Effects of GSSSG alone on cell viability and intracellular supersulfide levels in NHEKs. NHEKs were treated with 50 μM GSSSG in the absence of UVB irradiation. Data are presented as the mean ± standard deviation (SD). Statistical comparisons with the control group were performed using Student's t-test (***p < 0.001). (a) Cell viability determined by the MTT assay. No significant difference was observed between the control and GSSSG-treated groups. (b) Intracellular supersulfide levels. GSSSG treatment significantly increased intracellular supersulfide levels compared with those in the control group.

(a)

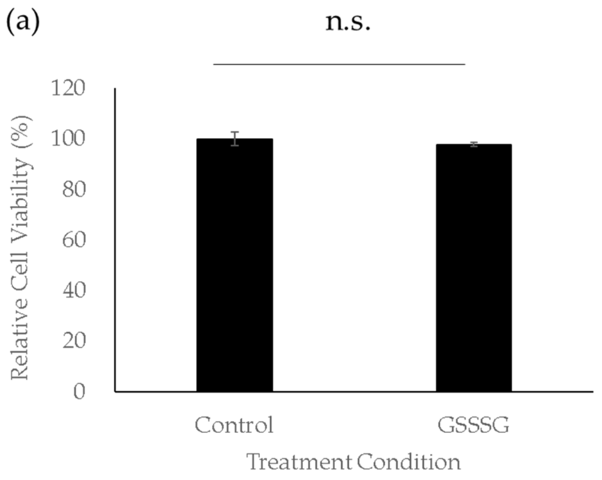

(b)

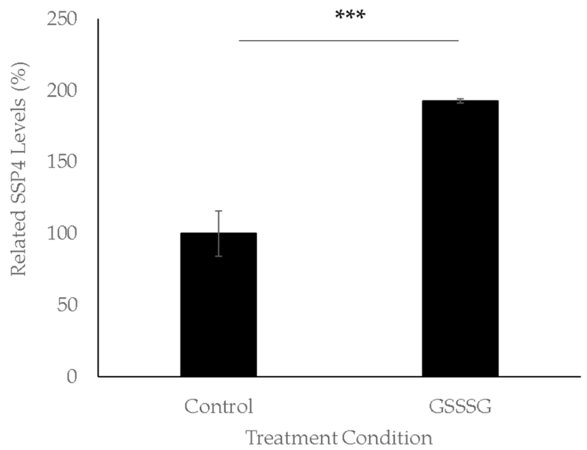

Figure S2. Representative images of NRF2 nuclear translocation in NHEKs. (a) Representative immunofluorescence images showing NRF2 localization in control, UVB-irradiated, and UVB + GSSSG-treated NHEKs. NRF2 is shown in green, and nuclei were stained with Hoechst (blue). Merged images are shown to visualize NRF2 nuclear

translocation. Scale bar = 25 μm. (b) Representative immunofluorescence images showing NRF2 localization in siControl-, siCARS2-, and siCARS2 + GSSSG-treated NHEKs following UVB irradiation.

(a)

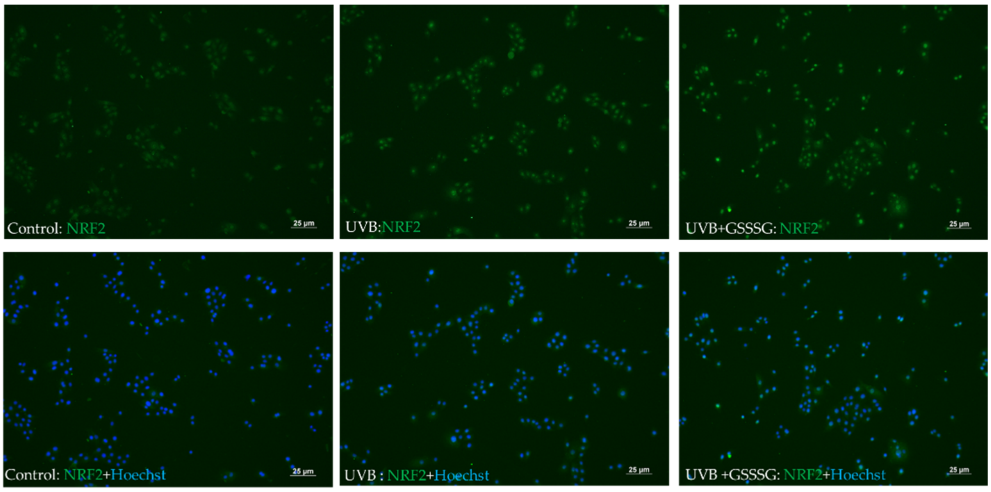

(b)

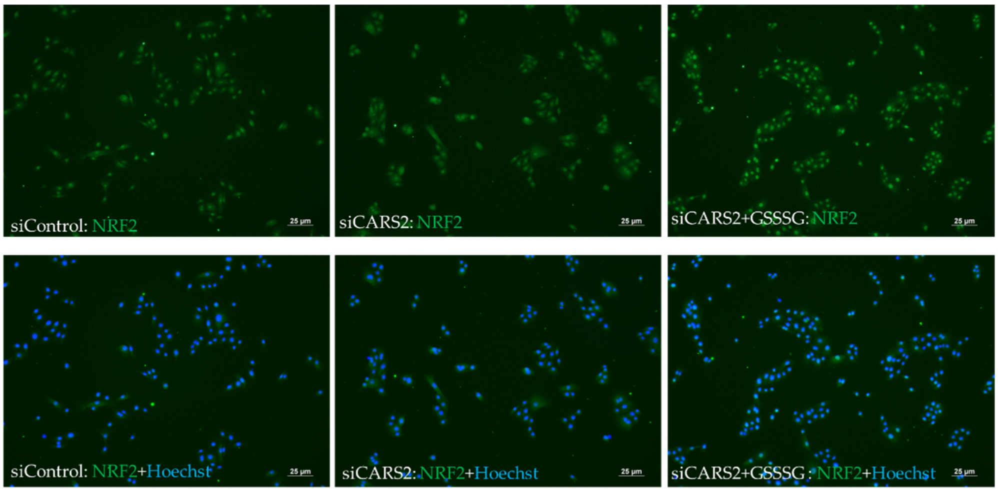

Figure S3. Validation of CARS2 knockdown in NHEKs. CARS2 mRNA expression was measured by RT-qPCR after transfection with control siRNA (siControl) or CARS2-targeting siRNA (siCARS2). Relative CARS2 mRNA levels were normalized to β-actin and expressed relative to the siControl group. Data are presented as mean ± SD (n = 3). Statistical significance was determined using Student's t-test. ***$p < 0.001$ vs. siControl.

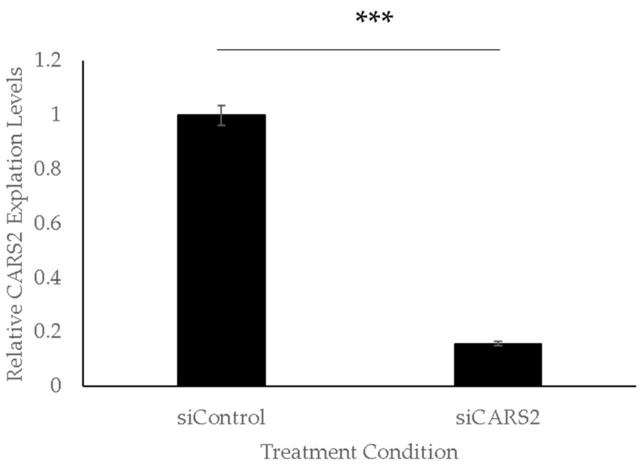
***
1.2
1
0.8
0.6
0.4
0.2
0
Relative CARS2 Explation Levels
siControl
siCARS2
Treatment Condition
